# Linking Only *Aedes aegypti* with Zika Virus Has World-Wide Public Health Implications

**DOI:** 10.3389/fmicb.2017.01248

**Published:** 2017-07-07

**Authors:** Fiona F. Hunter

**Affiliations:** Centre for Vector-borne Diseases, Department of Biological Sciences, Brock UniversitySt. Catharines, ON, Canada

**Keywords:** Zika virus (ZIKV), mosquito surveillance, vector competence, *Aedes aegypti*, *Culex quinquefasciatus*, Flaviviridae evolution, phylogenetics

## ZIKV mosquito vectors are not well-established

Zika virus (ZIKV) is an emerging arbovirus in the Americans and as such, we know far less about the mosquito species involved in transmission than many experts, public health authorities, and politicians would have the public believe.

The entomological literature with respect to ZIKV and the supposedly pivotal role that *Aedes aegypti* plays in its transmission is frighteningly scant. Multiple mosquito species have tested positive for the virus in field-collected specimens, including 20 species from genus *Aedes* (in alphabetical order, *A. aegypti, Aedes africanus, Aedes apicoargenteus, Aedes dalzieli, Aedes dorsalis, Aedes flavicollis, Aedes fowleri, Aedes furcifer, Aedes hirsutus, Aedes jamoti, Aedes luteocephalus, Aedes metallicus, Aedes minutus, Aedes neoafricanus, Aedes opok, Aedes taeniarostris, Aedes tarsalis, Aedes taylori, Aedes unilineatus, Aedes vittatus)*, as well as six non-*Aedes* species (*Anopheles coustani, Anopheles gambiae, Mansonia uniformis, Eratmapodites inornatus, Eratmapodites quinquevittatus*, and *Culex perfuscus*) (McCrae and Kirya, 1982; Haddow et al., 2012; Diallo et al., 2014).

In Malaysia, 58 pools of 1,277 *A. aegypti*, 59 pools of 4,492 *Aedes albopictus*, and 179 pools of 27,636 mosquitoes from 23 other *Aedes* species were surveyed for ZIKV and only a single pool of *A. aegypti* tested positive (Marchette et al., 1969). This was the first isolation of ZIKV outside of Africa (strain P6-740). In Gabon, 137 pools of 2,701 *A. albopictus*, 45 pools of 881 *A. aegypti*, 15 pools of 88 *Aedes simpsoni* complex, 29 pools of 690 *Culex quinquefasciatus*, and 21 pools of another 305 mosquitoes (made up of *An. gambiae, Mansonia africana, M. uniformis, Culex* sp., and *E. quinquevittatus*) were surveyed and two *A. albopictus* pools tested positive for ZIKV (Grard et al., 2014).

From the outset of the ZIKV outbreak in Brazil, the World Health Organization and other authorities have stated that *A. aegypti* is the mosquito that needs to be targeted for control in order to avoid a ZIKV pandemic; sometimes, *A. albopictus* is also included in the warnings. Novel strategies for controlling *A. aegypti* using *Wolbachia*-infected males or Oxitec® females (Yakob and Walker, 2016) target only *A. aegypti*. If ZIKV transmission is not being driven by *A. aegypti*, then these strategies will fail to protect people from ZIKV.

In mosquito surveys that followed the ZIKV outbreak on Yap Island in the Federated States of Micronesia, several species of mosquitoes were collected and tested for ZIKV; none was positive. This included a single negative *A. aegypti* specimen as well as 362 *Aedes hensilli*, the most abundant species, and 247 *C. quinquefasciatus*, the second most abundant (Ledermann et al., 2014). Subsequently, *A. hensilli* was experimentally infected with ZIKV MR766 (the original African lineage strain) and, despite relatively low dissemination rates, it was assumed that it served as a vector (Ledermann et al., 2014).

However, Guerbois et al. (2016) were able to collect mosquitoes during the 2015 ZIKV outbreak in Chiapas State, Mexico. They tested 471 female *A. aegypti* mosquitoes in 55 pools of which 15 were positive for ZIKV by RT-PCR. The second most abundant species was *C. quinquefasciatus* but no pools tested positive for ZIKV. Unfortunately, Guerbois et al. (2016) were only able to isolate virus from 3 of the 15 RT-PCR-positive samples following inoculation onto VERO cells.

Taken together, these data suggest a role for *A. aegypti* in the transmission of ZIKV but the data also leave room for other mosquito species (and other non-vector modes of transmission) to contribute to ZIKV outbreaks.

## Transmission studies involving *A. aegypti* and *A. albopictus*

In the 1950's *A. aegypti* mosquitoes were fed a blood-meal containing a ZIKV dose of 10^6.7^ mouse LD50 per 0.03 mL. There was one successful ZIKV transmission after a group of three infected mosquitoes fed on a rhesus monkey. The authors concluded “until it can be shown that *A. aegypti* can be infected with lower virus doses than those used here, its efficiency as a vector of Zika virus under natural conditions remains uncertain” (Boorman and Porterfield, 1956).

In another study, *A. aegypti* mosquitoes were infected intrathoracically with ZIKV ArD 24280 (an African lineage ZIKV strain isolated from *A. luteocephalus* in 1976) and subsequently the mosquitoes transmitted ZIKV to suckling mice (Cornet et al., 1979). However, for transmission to occur naturally, virus must be ingested during a blood-meal, cross over the midgut epithelium into the hemolymph, disseminate throughout the body, and eventually cross the salivary gland epithelium into the gland's lumen. Then, as a mosquito feeds on a subsequent blood-meal host, she spits saliva (containing virus along with anticoagulants, vasodilators, and salivary peptides) into the host. The experiment with ArD 24280 demonstrated that there was no salivary gland barrier, but it shed no light on the issue of a potential midgut barrier; thus, the study did not provide evidence that *A. aegypti* is a competent vector in the wild. However, the study did compare the ability of *A. aegypti* to transmit YF vs. ZIKV (both via intrathoracic infection) and found that the incubation period for ZIKV was less than for YF. This would suggest that *A. aegypti* is likely an efficient ZIKV vector.

A research group from Singapore infected *A. aegypti* using a blood-meal with ZIKV MR766 at an initial infectious dose of 7.0 Log^10^ tissue culture infectious dose_50_ (TCID_50_/mL). They observed both high infection rates and high salivary gland dissemination rates (Li et al., 2012). Unfortunately, saliva was neither collected nor tested so conclusions about transmission could not be made. The same group then conducted very similar experiments with *A. albopictus* but this time they collected saliva and tested it for virus. They found that on day 10 post-infection 100% of mosquitoes (*n* = 12) had ZIKV in their saliva (Wong et al., 2013). These studies provide support for the assumption that *A. albopictus* (and probably *A. aegypti*) are good vectors of ZIKV MR766. Nevertheless, the infection, dissemination and transmission rates reported in the two studies are exceptionally high compared to what other researchers have found. Although mosquito strain differences and/or viral strain differences might cause these differences, it is possible that mosquito husbandry techniques also may have played a role. During extrinsic incubation, the mosquitoes were fed “10% sugar/vitamin B complex *ad libitum*.” Most (if not all) mosquito transmission studies use only an *ad libitum* sugar meal (although concentrations may vary) for daily maintenance of mosquitoes. It is possible that the addition of vitamin B complex to the sugar meal may be partially responsible for the high infection, dissemination, and transmission rates.

Four *Aedes* spp. from Senegal—*A. aegypti, A. unilineatus, A. vittatus*, and *A. luteocephalus*—were tested for their potential to transmit African lineage ZIKV isolates (MR766 and HD78788) in the lab. All four species were infected orally. ZIKV-positive saliva was only detected from *A. vittatus* and *A. luteocephalus*. Despite relatively high infection rates in *A. aegypti*, dissemination rates were low (6.3% of 111 and 5.6% of 216 specimens from two different populations) and subsequent transmission rates (i.e., virus in saliva) were zero (Diagne et al., 2015).

Chouin-Carneiro et al. (2016) looked at the susceptibilities of *A. aegypti* and *A. albopictus* to an Asian lineage ZIKV strain (NC-2014-5132), fed at 10^7^ TCID_50_/mL. Similar to the Senegalese study (Diagne et al., 2015), authors found high infection rates but low dissemination and transmission rates for both species. Calculated transmission efficiencies were 3.3 ± 3.3% for *A. albopictus* and 10 ± 5.5% for *A. aegypti*. The authors concluded that “*Ae. aegypti* and *Ae. albopictus* were unexpectedly low competent vectors for ZIKV” (Chouin-Carneiro et al., 2016).

Aliota et al. (2016) studied the vector competence of *A. aegypti. A. albopictus, Aedes triseriatus*, and *Culex pipiens* fed on mice that had been infected with an Asian strain of ZIKV (PRVABC59). After 14 days' incubation, neither *A. triseriatus* nor *C. pipiens* had a disseminated infection or yielded virus in saliva. As positive controls, the researchers used *A. aegypti* and *A. albopictus*. They were able to detect virus in the saliva in 4 of 17 (24%) *A. aegypti* fed 6.83 log_10_ PFU/mL and in 2 of 9 (22%) *A. albopictus* fed 6.02 log_10_ PFU/mL.

In a recent study, Jupille et al. (2016) tested the vector competence of two populations of *A. aegypti* (from the island of Madeira) and two populations of *A. albopictus* (from France) using a ZIKV strain from New Caledonia (NC-2014-5132). They concluded that neither species was very susceptible to ZIKV. Virus was detected in the saliva of 1 of 20 (5%) *A. aegpyti* from Funchal and 0 of 20 (0%) *A. aegypti* from Paul do Mar on day 9 post-infection; data were identical for *A. aegpyti* on day 14. In contrast, the saliva of *A. albopictus* was negative for ZIKV for all specimens tested on day 9 from both Nice (*n* = 24) and Bar-sur-Loup (*n* = 24) and for *A. albopictus* tested on day 14 from Nice (*n* = 24). The saliva from only 1 of 24 (4.2%) *A. albopictus* from Bar-sur-Loup was positive on day 14.

Richard et al. (2016) tested the vector competence of two species implicated in 2013–2014 ZIKV outbreak in French Polynesia, using ZIKV strain PF13/251013-18. They found that “transmission efficiency was poor in *A. aegypti*” until 14 days post-infection and that *A. polynesiensis* was unable to transmit ZIKV at all. They concluded that there might be the “possible contribution of another vector for the propagation of ZIKV during the outbreak.”

Ayres (2016) cautioned about placing too much emphasis on *A. aegypti* in the battle against ZIKV, and suggested that *C. quinquefasciatus* might be an important vector in Recife, Brazil. Huang et al. (2016) looked at the vector competence of colonized *C. pipiens* and *C. quinquefasciatus* but were unable to demonstrate infection or dissemination at 7 or 14 days post-infection in mosquitoes held at 28°C. Fernandes et al. (2016) were unable to show transmission in *C. quinquefasciatus*.

The first published report of the vector competence of *C. quinquefasciatus* comes from Guo et al. (2016). They were able to demonstrate that mosquitoes held at 29°C could transmit ZIKV by bite to suckling mice and furthermore, that the peak time of virus appearance in the salivary glands was day 8 post-infection.

With all of the conflicting reports in the literature, it is prudent to consider other evidence that might shed light on which mosquito species are involved in ZIKV transmission.

## Evidence from phylogenetics

**Figure 1:**
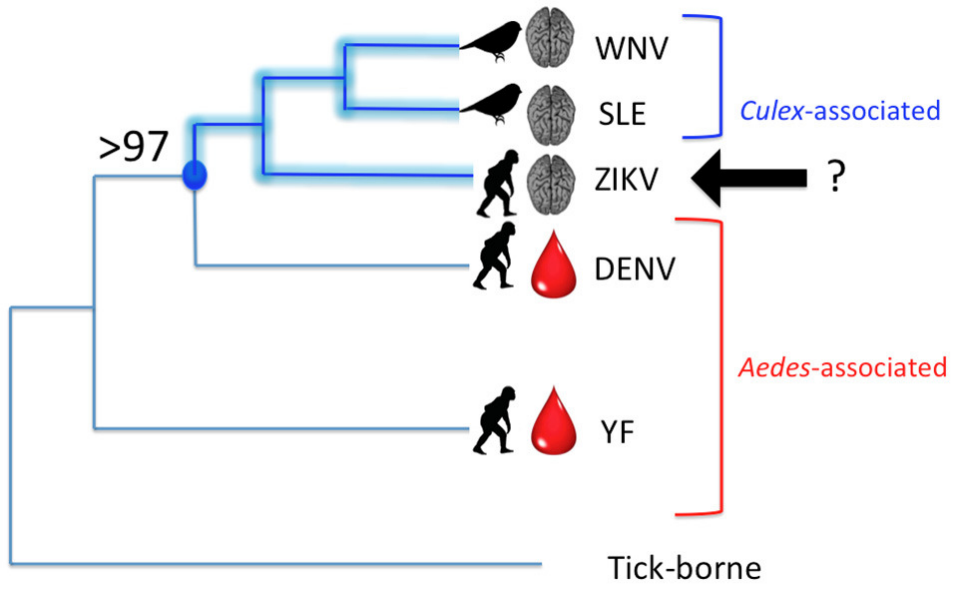
Phylogenetic relationships among several mosquito-borne Flaviviruses. WNV, West Nile virus; SLE, Saint Louis Encephalitis virus; ZIKV, Zika virus; DENV, Dengue virus; YF, Yellow fever virus. Lengths of branches do not reflect phylogenetic distances, but tree topology is in agreement with several published phylogenies, including Moureau et al. (2015). Brackets indicate “*Culex*”-associated and “*Aedes*”-associated viruses, in keeping with the ICVT, but with ZIKV unassigned. A crucial node (indicated in blue) is supported by 97–100% of molecular analyses of the *Flavivirus* genome. This node defines the clade of [ZIKV + [SLE + WNV]]. Disease indicators are overlaid on the phylogeny with a brain representing neurotropic effects and a drop of blood, hemorrhagic effects. Known reservoir hosts are shown with either a bird silhouette or a primate silhouette.

There is an additional line of evidence—the evolutionary history of the Flaviviridae—that points to species other than *A. aegypti* as playing key roles in the current ZIKV outbreaks. Numerous authors have reconstructed phylogenies of the Flaviviridae based on amino acid sequences and nucleotide sequences. Figure 1 is a synthesis of three papers (Kuno et al., 1998; Lanciotti et al., 2008; Moureau et al., 2015). According to the International Commission on Viral Taxonomy (ICVT), West Nile virus (WNV), and Saint Louis Encephalitis virus (SLE) are designated as “*Culex*-associated” viruses whereas Dengue virus (DENV), Yellow Fever virus (YF), and ZIKV are “*Aedes*-associated” viruses (see also Moureau et al., 2015). The “*Culex*-associated” flaviviruses are known for their bird reservoirs and human neurotropic effects (e.g., encephalitis and paralysis) whereas “*Aedes*-associated” flaviviruses such as Dengue virus (DENV) and Yellow Fever virus (YF) are known for primate reservoirs and hemorrhagic diseases.

Phylogenetically, ZIKV clearly belongs to the lineage that contains WNV and SLE, two so-called “*Culex*-associated” viruses. Nodal support for the [ZIKV + [SLE + WNV]] clade ranges from 97 to 100% (Kuno et al., 1998; Lanciotti et al., 2008; Moureau et al., 2015). However, several of these same authors still consider ZIKV to be *Aedes*-associated according to convention. Based on the phylogenetic relationships, the most parsimonious interpretation is to include ZIKV within the *Culex*-associated lineage. This is in line with comments made by Grard et al. (2010) who analyzed the complete coding sequence of *Aedes*-borne flaviviruses [*sensu* ICVT] and concluded that, based on an analysis of amino acid distances in the NS5 gene, ZIKV is “clearly related to *Culex*-borne flaviviruses.” Using this logic, the fact that ZIKV is linked to neurotropic effects—such as fetal brain abnormalities and Guillain-Barré syndrome—comes as no surprise. Due to the relatively low number of human ZIKV cases prior to 2007, severe neurotropic symptoms were previously unknown, and were unexpected due to the paradigm that ZIKV was a traditional “*Aedes*-borne” virus [*sensu* ICVT] akin to DENV.

Figure 1 also shows the association with disease indicators, namely neurotropic effects (indicated by a brain) or hemorrhagic effects (indicated by a drop of blood). The common vertebrate reservoirs are also indicated with silhouettes of either birds or non-human primates. In terms of disease indicators, ZIKV is more like WNV and SLE, but possibly more like DENV and YF in terms of reservoir hosts. Or is it? A forgotten paper by Okai et al. (1971) reported that 15% of 221 birds collected in Uganda tested positive for ZIKV antibodies by Hemagglutination Inhibition Assays, with the majority of positive birds being Greenbuls (Family Pycnonotidae). The Okai et al. (1971) study would support the addition of a bird silhouette to the ZIKV branch in Figure 1.

## Keep an open mind

Scientists need to consider the possibility that ZIKV may be more similar to the classic “*Culex*-associated” flaviviruses than it is to the “*Aedes*-associated” viruses by collecting and testing field-collected *Culex* spp., by screening a number of different vertebrates for their potential roles as reservoirs, and by studying the vector competence of *Culex* spp. in the laboratory. Aliota et al. (2016) have reported that colony-reared *C. pipiens* were unable to become infected with ZIKV strain PRVABC59. Weger-Lucarelli et al. (2016) also showed that colony-reared *C. pipiens, C. quinquefasciatus*, and *C. tarsalis* were refractory to ZIKV strain PRVABC59. However, additional studies on the vector competence of *Culex* mosquitoes to other ZIKV strains need to be conducted and published. In certain geographic regions, *C. quinquefasciatus* is a highly peridomestic mosquito and it feeds avidly on humans; furthermore, it has been found in relatively large numbers when mosquito surveillance has been conducted for ZIKV (Grard et al., 2014: Gabon, third most abundant species; Ledermann et al., 2014: Yap, second most abundant species). The World cannot afford to concentrate all of its efforts on monitoring and controlling *A. aegypti* in the fight against ZIKV, especially if there are *Culex* mosquitoes that may also serve as competent vectors.

Consideration ought to be given to the phylogenetic evidence that “*Culex*-borne” flaviviruses have evolved from ancestral “*Aedes*-borne” flaviviruses (Grard et al., 2010) and that means that an expanded (rather than a restricted) vector range might be expected for ZIKV. Furthermore, based on a data-driven model linking mosquito vector species and vector-virus traits, Evans et al. (2017) have predicted that as many as 35 different mosquito species could be vectors for ZIKV.

Medical entomologists, public health professionals, and politicians are urged to keep an open mind on the issue of which mosquito species need to be targeted for control in the battle against ZIKV.

## Author contributions

The author confirms being the sole contributor of this work and approved it for publication.

### Conflict of interest statement

The author declares that the research was conducted in the absence of any commercial or financial relationships that could be construed as a potential conflict of interest.
